# Predictive factors of diabetic complications: a possible link between family history of diabetes and diabetic retinopathy

**DOI:** 10.1186/2251-6581-13-55

**Published:** 2014-05-08

**Authors:** Zhila Maghbooli, Parvin Pasalar, Abbasali Keshtkar, Farshad Farzadfar, Bagher Larijani

**Affiliations:** 1 Endocrinology and Metabolism Clinical Sciences Institute of Tehran University of medical sciences, Postal address; EMRI, 5th floor, Shariati Hospital, North Karegar avenue, P.O Box: 1411413137, Tehran, Iran; 2 Biochem, Department Faculty of Medicine Tehran University of medical sciences, Postal address; EMRI, 5th floor, Shariati Hospital, North Karegar avenue, P.O Box: 1411413137, Tehran, Iran; 3 Endocrinology and Metabolism Population Sciences Institute of Tehran University of medical sciences, 4th floor, No. 4, Ostad Nejatollahi St, Enqelab Ave, 1599666615 Tehran, Iran

**Keywords:** Diabetic retinopathy, Epigenetic, Family history of diabetes

## Abstract

**Background:**

The aim of this study was assessment of predictive factors of diabetic retinopathy.

**Methods:**

A cross-sectional study was designed by recruiting 1228 type 2 diabetic patients from a diabetes referral clinic over a six-month period (from July to December, 2012). Diabetes risk factors, complications, laboratory results have been recorded.

**Results:**

Of the 1228 diabetic patients (54% women, mean age 58.48 ± 9.94 years), prevalence of diabetes retinopathy was 26.6%. There were significant associations between retinopathy and family history of diabetes (p = 0.04), hypertension (p = 0.0001), diabetic duration (p = 0.0001), poor glycemic control (p = 0.0001) and age of onset of diabetes (p = 0.0001). However, no significant associations were found between retinopathy with dyslipidemia and obesity. In logistic regression model, poor glycemic control (p = 0.014), hypertension (p = 0.0001), duration of diabetes (p = 0.0001) and family history of diabetes (p = 0.012) independently predicted retinopathy after adjustment for age and sex.

**Conclusions:**

Diabetic complications are resulting from an interaction from genes and environmental factors. A family history of diabetes is pointing toward a possible genetic and epigenetic basis for diabetic retinopathy. Our findings suggest the role of epigenetic modifications and metabolic memory in diabetic retinopathy in subjects with family history of diabetes.

## Background

Global epidemic of diabetes is a serious and a major health care concern that results in reduced life expectancy and increased morbidity due to disease-specific micro- and macro vascular complications. The strongest risk factors for diabetic micro vascular complications are poor glycemic control and diabetes duration. It is notified, despite good glycemic control, vascular complications remain in most diabetic patients [1,2]. In addition, diabetic complications may develop before diagnosis [3,4].

Although it is accepted that diabetic complications result from abnormal metabolic environment engendered by chronic hyperglycaemia, the risk of developing these complications is under the control of *genetic* factors. But efforts to identify genetic variants have produced disappointing. A family history of type 2 diabetes has been suggested as a strong link to genetic factors. Genetic susceptibility to diabetes and its complications was supported by some linkage and familial aggregation studies. This evidence suggests that genetic contributions may influence the development of vascular complications [5-7]. Genome wide association studies demonstrated some genetic variations could explain inter-individual variations in the susceptibility to diabetic complications [8-10]. However, in the absent of specific genes or loci that show strong relationship with diabetic vascular complications, several of clinical and epidemiological studies have demonstrated that environmental factors as well as genetic factors play an etiological role for onset of diabetic vascular complications [11,12]. Familial clustering of diabetic complications could result from shared genes, environmental exposures, or their combination. However, the exact nature of these two main etiological factors and their pathogenic mechanisms are poorly understood. Increasing knowledge about predictive factors of diabetic complications causes that they will be preventable in diabetic patients. Early identification of risk factors can help reduce the development and progression of diabetic micro vascular complications, and improve patients' quality of life.

Individual SNPs may confer risk of vascular complications [13,14] but they are not useful as a predictive factor for onset of complications and not clinically informative [15]. Therefore, this study has been intended assess predictive factors of diabetic micro vascular complications that is caused by a combination of genetic and environmental factors. For this aim, we have chosen retinopathy that is the most common diabetic micro vascular complication. This cross-sectional study was designed to investigate prevalence of diabetic retinopathy (DR), risk factors, laboratory results, glycemic control and family history of diabetes in order to determine the role of each factor to predict DR.

## Methods

### Setting

A cross-sectional study was performed on 1228 adults with type 2 diabetes mellitus recruited from the diabetes referral clinic of the Tehran University of Medical Sciences over a six-month period (from July to December, 2012). The protocol for the research project has been approved by Ethics Committee of Endocrinology and Metabolism Research Institute of Tehran University of medical sciences. Patients affected by Type 1 diabetes were excluded from the study. The diagnosis of diabetes type 2 was carried out and/or confirmed following the American Diabetes Association criteria [16]; a fasting blood glucose of greater than or equal to 126 mg/dL on two separate occasions; a random (non-fasting) blood glucose of greater than or equal to 200 mg/dL on two separate occasions; or a blood glucose of greater than 200 mg/dL at 2 hours during a 75 gr oral glucose tolerance test.

Demographic character and clinical information including sex, age, age at diabetes diagnostic, diabetes duration, current use of anti-diabetic, antihypertension, and lipid-lowering medications, systolic and diastolic blood pressure, cigarette smoking status, body mass index (BMI), lipid profile, glycated haemoglobin (HbA1c), and present of diabetes complications (retinopathy, nephropathy, and coronary artery disease) were evaluated. Diabetic nephropathy was defined as persistent increased urinary albumin excretion (UAE) ≥300 mg/24 h or total proteinuria > 500 mg/24 h in the absence of other renal diseases. Coronary artery disease was defined as the presence of a visible luminal narrowing of 50% or more in at least 1 coronary artery detected by angiography.

### Retinopathy screening and diagnosis

Based on ADA guideline, annual eye examinations were performed for all diabetic patients, for retinopathic patients each 6 month (by dilating the pupils with eye drops and then carefully examining the retina) by an ophthalmologist. Fluorescein angiography was performed at risk patients.

### Definition of diabetes major risk factors

Hypertension was defined in subjects with a BP > 140/90 mmHg or taking BP medication. Based on ADA criteria dyslipidemia was defined as triglycerides (TG) > 250 mg/dl and/or high-density lipoprotein (HDL) < 35 mg/dl [17]. Glycemic control was categorized into poor and good glycemic control based on HbA1c ≥ 7% or HbA1c < 7%, respectively. Obesity was classified based on BMI ≥30 kg/m^2^.

### Statistical analysis

All statistical analyses were carried out using SPSS, version 16. A descriptive analysis of all available variables was performed. Logistic regression model was used to examine associations of potential risk factors with diabetic retinopathy. Numerical variables are reported as the mean ± standard deviation and categorical variables are presented as percentages. The level of statistical significance was 5%.

## Results

The baseline characteristics of the 1228 diabetic patients (54% women) are presented in Table 1. The prevalence of diabetes risk factors including dyslipidemia, hypertension, and obesity was 90.7%, 72.6%, and 35.7%, respectively. Poor control of glycemia was observed on 58.1% (642/1105) of patients.

**Table 1:** The baseline characteristics of the type 2 diabetic patients

**Characteristic**	**Type 2 diabetic patients (N = 1228) (mean ± SD)**
Age- (year)	58.48 ± 9.94
duration of diabetes- (year)	11.33 ± 7.98
Age of onset- (year)	47.34 ± 11.05
Glycated haemoglobin-%	7.5 ± 1.68
Total cholesterol— mg/dl	149.41 ± 32.7
LDL - mg/dl	86.15 ± 30.64
HDL- mg/dl	44.39 ± 12.14
Triglycerides — mg/dl	154.56 ± 91.87
Systolic Blood pressure-mm Hg	126.5 ± 20.41
diastolic Blood pressure- mm Hg	74.6 ± 11.32
BMI (Kg/m2)	28.75 ± 5.63

At least one diabetic complication (cardiovascular diseases, retinopathy, and nephropathy) was diagnosed in 477 diabetic patients (52.6%). The prevalence of diabetes nephropathy, cardiovascular diseases and diabetes retinopathy were 10.7%, 17.7%, and 26.6%, respectively.

The total of 1050 patients had ophthalmic data available for analyses. Among patients with retinopathy, the prevalence of non-proliferative diabetes retinopathy (NPDR), proliferative diabetes retinopathy (PDR), and clinically significant macular edema (in patients with and without PDR, NPDR) was 65.8%, 8.7% and 29.5%, respectively. Patients with retinopathy were tended to be older, with a longer duration of diabetes (p = 0.0001) and earlier mean age of onset of diabetes (p = 0.0001), (Table 2). Poor glycemic control was observed in 69.7% patients with retinopathy compare to 52.8% in patients without retinopathy (p = 0.0001). Among diabetic risk factors, there were significant associations between retinopathy and a family history of diabetes (OR = 1.59, 95% CI: 1.003 to 2.28, p = 0.04), and hypertension (OR = 1.60, 95% CI: 1.68 to 3.55, p = 0.0001). However, no significant associations were found between retinopathy with dyslipidemia (p = 0.41) and obesity (p = 0.54). The prevalence of retinopathy in patients with first and second degree family history of diabetes was significantly higher than patients without family history of diabetes; 26.2% (167/638), 37.7% (29/77), and 19.9% (32/161), respectively (p = 0.012).

**Table 2:** Comparison risk factors in patients with and without retinopathy

**Risk factors**	**Patients with retinopathy (n = 279)**	**Patients without retinopathy (n = 771)**	**Pvalue**
Age	60.35 ± 8.91	57.4 ± 10.05	0.0001
Sex (male)	47.0%	43.8%	0.37
Age of onset of diabetes	44.33 ± 10.55	48.2 ± 10.89	0.0001
Duration of diabetes	16.07 ± 8.44	9.39 ± 6.87	0.0001
Poor glycemic control	69.7% (184/264)	52.8% (374/708)	0.0001
hypertension	85.2% (236/277)	69.2% (529/765)	0.0001
dyslipidemia	93.9% (262/279)	91.2% (701/769)	0.15
Obesity	36.8% (75/204)	34.4% (217/630)	0.54

In diabetic patients with family history of diabetes, there was significant association between poor glycemic control and DR (p = 0.001). In contrast, in diabetic patients without family history of diabetes, there was not association between glycemic control and DR (p = 0.09).

Overall, diabetic patients with family history of diabetic had higher prevalence of hypertension (78.5% vs. 21.5%, 95% CI: 0.4 to 0.88, p = 0.009), and earlier age at onset (49.78 ± 11.73 vs. 46.64 ± 10.83) (95% CI: 1.4 to 4.8, p = 0.0001) than those without family history.

To investigate predictive factors of DR, a logistic regression model was used. In this model, after adjusting for age and sex, glycemic control (p = 0. 014, OR = 1.56), hypertension (p = 0.0001, OR = 2.33), duration of diabetes (p = 0.0001, OR = 4.27) and family history of diabetes (p = 0.012, OR = 1.41) were independent predictors of retinopathy. In this model, there were not significant relationships between obesity (p = 0.54), and dyslipidemia (p = 0.10).

## Discussion

Diabetic retinopathy is most severe microvascular complication in patients with Type 2 Diabetes which leading cause of vision loss in working-aged adults (20–74 years) [18]. High prevalence of DR in type 2 diabetic patients imposes a large economic burden and public health concern on the national healthcare system [18]. In our study the prevalence of DR in patients with type 2 diabetes was 26.6%. Our study has clarified that poor glycemic control, duration of diabetes, hypertension and family history of diabetes are the most important predictors of DR. The underlying causes and prediction factors of DR have not been completely elucidated. Severity of hyperglycemia, present of hypertension and diabetes duration; are widely recognized as major risk factors for the development of DR [19,20]; however explained approximately 10% of variation in DR [21-23].

**Figure 1:**
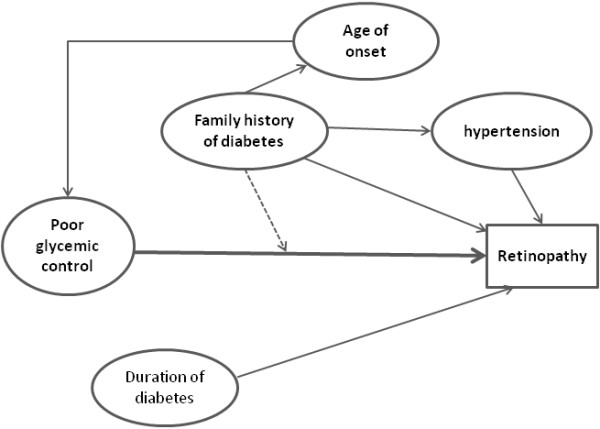
Model proposing a role for retinopathy risk factors in related to each other.

Epidemiological studies have suggested primarily intensive glycemic control can delay the development of DR [19,20]. It is however noteworthy that some patients may still develop DR even with good glycemic control. Although our result has shown duration of diabetes to be related to development of DR, it may begin to develop as early as 7 years before the diagnosis of diabetes in patients with type 2 diabetes [4]. Our finding has showed that family history of diabetes is an independent predictor of DR. In addition; a family history of diabetes synergistically modifies association between poor glycemic control and risk of DR development (Figure 1). Our results confirmed with sibling, twin and family studies. Heritability scores with ranging from 27-52% in both type 1 and type 2 diabetes were reported in these studies and also retinopathy aggregates in family studies [7,24-26].

Although studies of familial aggregation suggested that genetic factor influence on onset of DR or its severity, genetic studies reported conflicting results [25,26]. Several candidate genes and loci were associated with DR. However, few of them have been replicated [27], with weak genetic associations [13,14,28]. In addition, genetic variations only were associated with a small proportion of the total phenotypic variation, even for conditions that are known to be highly heritable [29]. Therefore, a family history of diabetes suggests familial genetic and epigenetic contributions to the disease complications.

*Epigenetics* is a newer area of research beginning to make clear gene-environmental interactions and to identify factors that may alter gene expression and function across the lifespan. Epigenetic processes may be particularly important for understanding complex disorders like diabetes because they are consistently heritable despite having relatively weak and inconsistent association with individual genetic variants. There is growing evidence that gene-gene interactions and gene-environmental interactions play an important role in determining an individual’s risk of various common diseases including diabetes [30]. Recent evidence shows that most gene expressions and gene-environmental interactions are mediated by epigenetic modulations [31-33]. Epigenetic mechanisms provide a framework to incorporate environmental factors into models of complex disorder risks.

In particular, it has been noted that some individuals with diabetes experience a continued progression of vascular complications even after glycemic control subsequent to a period of prior hyperglycaemic exposure, a phenomenon termed ‘metabolic memory'. “Metabolic Memory”, is the idea that early glycemic environment is remembered in the target organs [34] which has been demonstrated in large multiple clinical trials [19,35]. These studies demonstrated that intensive glycemic control could reduce the progression of diabetic complications but could not prevent them. Compelling evidence exists suggesting that exposure to an adverse fetal and/or early postnatal environment may enhance susceptibility to a number of chronic diseases in the future life of offspring. Furthermore, this evidence supports epigenetic mechanisms as important components in metabolic memory and the pathology of diabetic complications [32]. But this hypothesis remains to be confirmed.

## Abbreviations

DR: Diabetic retinopathy; TG: Triglycerides; HDL: High-density lipoprotein.

## Competing interests

The authors declare that they have no competing interest.

## Authors’ contributions

All authors designed the study. ZhM gathered the clinical data. AAK, FF and ZhM analyzed the data. ZhM, PP, AAK and BL wrote the main paper. All authors discussed the results and commented on the manuscript at all stages. All authors read and approved the final manuscript.
